# PD35 Tailored Health Technology Assessment Capacity Building: Co-Designing Training Resources For Patients And Caregivers In Singapore

**DOI:** 10.1017/S0266462325101694

**Published:** 2025-12-29

**Authors:** Jen Hun Koh, Sok Huang Teo, Fiona Pearce, Ping-Tee Tan

## Abstract

**Introduction:**

In Singapore, patient involvement has been an integral component of health technology assessment (HTA) since 2022. Processes co-designed with local patient organizations have encouraged meaningful collaboration, reflection, and learning. This presentation discusses the impact of tailored training resources implemented by the Agency for Care Effectiveness (ACE) to build capacity and empower patients and caregivers to contribute to healthcare decision-making.

**Methods:**

Surveys were electronically distributed to 27 local patient organizations in 2023 to identify knowledge gaps and the information needs of their members. A comprehensive review of patient resources from overseas HTA agencies and patient groups was undertaken to identify best practices for enhancing capacity building. The insights gathered informed the co-development of two interactive training workshops and a series of self-directed learning modules on HTA. Feedback was gathered from participants at the end of each workshop, and post-training surveys were conducted to assess changes in participants’ understanding of HTA, skill development, attitudes towards patient involvement, and expectations for future training resources.

**Results:**

Thirty-seven patient leaders attended the workshops, which introduced HTA in plain language and role-playing as decision-makers to explore factors considered for funding recommendations. Learning modules were published online and disseminated to all local patient organizations. Survey results showed that the training resources led to marked increases in patients’ self-reported confidence to participate in future HTAs, and in their knowledge of HTA concepts. The resources were viewed as a positive step toward improving the quality of patient input in HTAs, and overcoming local perceptions of tokenistic involvement, by strengthening the value of experiential knowledge over traditional scientific evidence.

**Conclusions:**

Workshops and learning modules tailored specifically to the information needs of patients play an important role in building confidence and skills in HTA. ACE will continue to expand its resources to equip patients and caregivers with the skills required to meaningfully participate in healthcare decision-making. Impact evaluation of training initiatives will drive continuous improvement and ensure they remain relevant to patients’ needs.

